# Gallbladder neuroendocrine carcinoma: A single center experience

**DOI:** 10.1097/MD.0000000000021912

**Published:** 2020-09-04

**Authors:** Hongwu Chu, Chengwu Zhang, Ying Shi, Weiding Wu, Zhiming Hu, Jungang Zhang, Dongsheng Huang

**Affiliations:** a Hepatobiliary and Pancreatic Surgery; b Qingdao University, Qingdao; c Obstetrics and Gynecology, Zhejiang Provincial People's Hospital, Hangzhou Medical College, Hangzhou, China.

**Keywords:** clinical feature, gallbladder, neuroendocrine carcinoma, treatment

## Abstract

Gallbladder neuroendocrine carcinoma (GB-NEC) is a group of rare and heterogeneous neoplasms and there are few reports at present.

We analyzed the clinical and pathological features of 7 patients with GB-NEC who were admitted to Zhejiang Provincial People's Hospital from January 2011 to October 2019.

The median age of 7 patients was 58 years with male to female ratio of 1:2.5. Right upper quadrant discomfort was the main complaint and no patients presented carcinoid syndrome-related symptoms. In contrast-enhanced computed tomography (CT) examination, 5 of 6 patients showed well-defined margin and continuous thin line-like contrast enhancement on the mucosa. Among the patients with liver metastases before surgery, 66.7% of patients were cancer antigen 125 (CA-125) positive, and among the patients presented with liver metastases during follow-up period, all patients were CA-125 positive. All patients with elevated CA-125 did not have ascites, ovarian carcinoma, peritoneal carcinoma, and endometrial carcinoma. According to postoperative pathological report, 1 patient was stage IIIA, and the other 6 patients were stage IVB. Six patients underwent surgery, and 1 patient just underwent liver biopsy. Two patients underwent laparoscopic radical cholecystectomy, and neither of them encountered serious complications after surgery with the overall survival time of 4.6 and 16.8 months, respectively. Compared with the patients without chemotherapy, 3 patients postoperatively treated with chemotherapy lived longer. The median survival of all 7 patients was 4.6 months and the 1-, 2-year survival rates were 14.29%, 0%.

Surgical resection, including laparoscopic radical cholecystectomy, is feasible for the treatment of advanced GB-NEC in selected patients and has the advantages of prolonging survival in combination with chemotherapy. The elevation of CA-125 can be utilized as an important predictor of poor prognosis, while more investigations are necessary to confirm it.

## 1 Introduction

Neuroendocrine neoplasms (NENs) are recognized as a group of rare and heterogeneous tumors that originate from disseminated neuroendocrine cells throughout the whole body.^[1]^ According to the Surveillance, Epidemiology and End result (SEER) database, the incidence of NENs in America was 6.98 per 100,000 in 2012.^[2]^ They are more common in gastrointestinal tract and respiratory tract, accounting for 66% and 31% of all NENs, respectively.^[3]^ Primary gallbladder neuroendocrine neoplasm (GB-NEN), which comprises only 0.5% of all NENs, is extremely rare in clinical practice with few reports. The data from the SEER database showed that the 1-year, 2-year, 3-year, and 4-year survival rates of GB-NEN in America were 43% to 45%, 30% to 33%, 28% to 31%, and 22% to 26%, respectively.^[4]^ According to 2019 World Health Organization (WHO) classification, gallbladder neuroendocrine carcinoma (GB-NEC) is defined as a kind of poorly differentiated NEN, and encompasses small-cell and large-cell type.^[5]^ Due to the fact that GB-NEC is lack of special clinical manifestations, typical imaging features, and tumor markers, it is difficult to preoperatively distinguish GB-NEC from other gallbladder carcinomas. Compared with gallbladder adenocarcinoma (GB-ADC), GB-NEC has different characteristics, and is often diagnosed at advanced stage and associated with a worse prognosis.^[6]^ However, due to the low morbidity, investigation regarding the pathogenesis, treatment modalities, and prognosis of GB-NEC is currently rare. In this study, we analyzed the clinical and pathological features of GB-NEC, and introduced our center's experience in the diagnosis and treatment of GB-NEC and the relationship between Cancer antigen 125 (CA-125). elevation and poor prognosis of GB-NEC.

## 2 Material and methods

### 2.1 Patients

Patients with GB-NEC who were treated at the Zhejiang Provincial People's Hospital from January 2011 to October 2019 were included in the study. The inclusion criteria were as followed: the primary tumor site was in the gallbladder; patients were diagnosed as GB-NEC by experienced pathologists based on typical morphological characteristics and immunohistochemical features, according to 2019 WHO classification^[5]^; the medical records and follow-up data of patients were detailed and intact. Finally, 7 patients were taken into analysis, and no patients were excluded. This study was conducted in accordance with the Declaration of Helsinki and approved by the Clinical Research Ethics Committee of the Zhejiang Provincial People's Hospital of Hangzhou, China.

### 2.2 Data collection

Clinical characteristics, imaging examinations, biochemistry examinations, tumor markers, treatment modalities, histopathological and immunohistochemical characteristics, postoperative days of oral diet recovery, postoperative days of drainage tubes removal, postoperative days of hospital stay, postoperative complications, and postoperative survival, were collected. The normal range of the liver function tests and infection tests were as followed: aspartate aminotransferase (AST): 13.0 to 35.0 U/L, alanine aminotransferase (ALT): 7.0 to 40.0 U/L, alkaline phosphatase (ALP): 35.0 to 100.0 U/L, gamma-glutamyl transferase (GGT): 7.0 to 45.0 U/L, albumin: 40.0 to 55.0 g/L, total bilirubin (TBIL): 3.4 to 24.0 μmol/L, prothrombin time (PT): 9.99 to 13.52 seconds, international normalized ratio (INR): 0.85 to 1.15, white blood cell (WBC): 3.5 to 9.5 × 10^9^/L, C-reactive protein (CRP): 0 to 10.0 mg/L. Above indicators beyond the normal range were considered positive. Cancer antigen 125 (CA-125) in here should be replaced with CA-125. > 35.0 U/mL, cancer antigen 199 (CA-199) > 37.0 U/mL, carcinoembryonic antigen (CEA) > 4.7 μg/L, alpha fetoprotein (AFP) > 20.0 μg/L was considered positive. The choice of treatment depended on preoperative imaging findings and intraoperative conditions, including local tumor infiltration, lymphatic metastasis, and distant metastasis. Bile leakage was according to the definition proposed by International Study Group of Liver Surgery.^[7]^

### 2.3 Pathological classification and staging

In this study, the diagnosis of GB-NEC was based on the 2019 WHO classification of tumors of the digestive system. Tumor-node-metastasis (TNM) stage was determined according to the American Joint Committee on Cancer (eighth edition), based on postoperative pathological report.^[8]^ According to the 2019 WHO classification, NENs are classified into 3 main categories: well-differentiated neuroendocrine tumors (NETs), poorly-differentiated NECs, and mixed endocrine non-endocrine neoplasms (MiNENs).^[5]^ Furthermore, NETs can be graded into G1, G2, and G3, based on mitotic rate and/or the Ki-67 index: G1, mitotic rate <2 per 2 mm^2^ and/or Ki-67 index ≤2%; G2, mitotic rate 2 to 20 per 2 mm^2^ and/or Ki-67 index 3% to 20%; G3, mitotic rate >20 per 2 mm^2^ and/or Ki-67 index >20%. NECs are poorly-differentiated and encompasses small-cell and large-cell type with mitotic rate >20 per 2 mm^2^ and/or Ki-67 index >20%. MiNENs are highly differentiated or poorly differentiated, consisting of endocrine and non-endocrine components, and the proportion of each component is >30%. Moreover, the non-neuroendocrine components of MiNENs are not necessarily all adenocarcinomas, but may also be squamous cell carcinomas.

### 2.4 Postoperative follow up

Abdominal ultrasound, contrast-enhanced computed tomography (CT) and/or magnetic resonance imaging (MRI), liver function test, and serum tumor markers level were performed every month for the first 3 months and then every 3 months for the initial 2 years. Telephone follow-up was conducted by a research nurse every month to update the survival status of patients. Overall survival time was defined from the date of operation to the date of death for any reason, or to the date of last follow-up in February 2020.

## 3 Results

### 3.1 Clinical characteristics

Two patients were men and 5 were women, with a male to female ratio of 1:2.5. The median age was 58 years (ranging from 37 to 79 years). The most common clinical symptom was right upper quadrant pain (5/7, 71.4%), followed by poor appetite (2/7, 28.6%), jaundice (1/7, 14.3%) (Table 1). No patient complained carcinoid syndrome-related symptoms such as diarrhea, edema, flushing, or wheezing. In addition, 3 patients had gallstones, 4 had hypertensions, and 1 had type II diabetes.

**Table 1:**
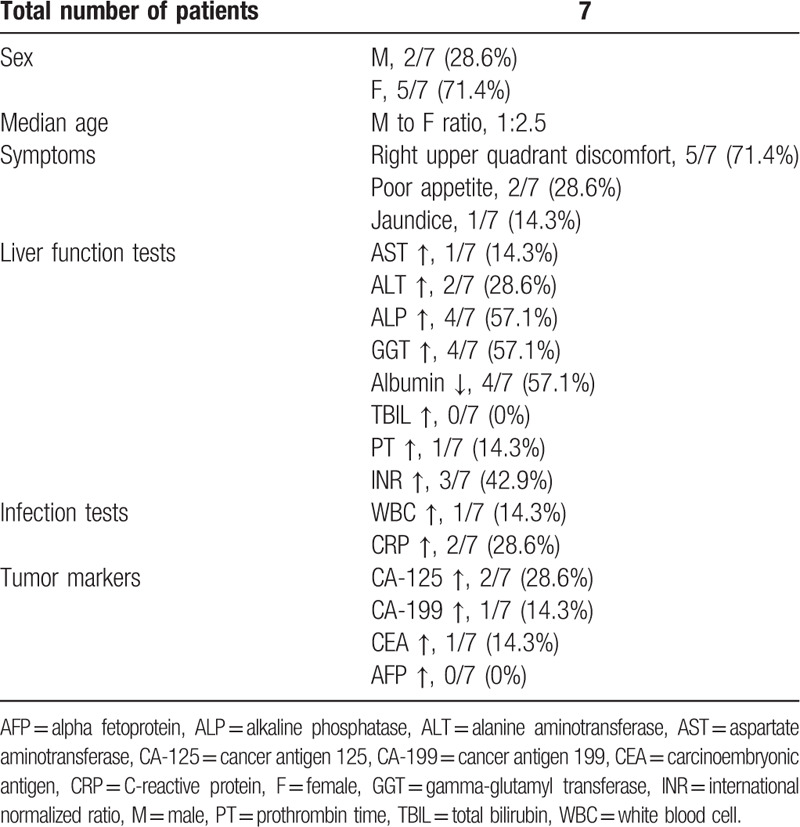
Characteristics of patients with gallbladder neuroendocrine carcinoma.

### 3.2 Imaging and laboratory examination

**Figure 1:**
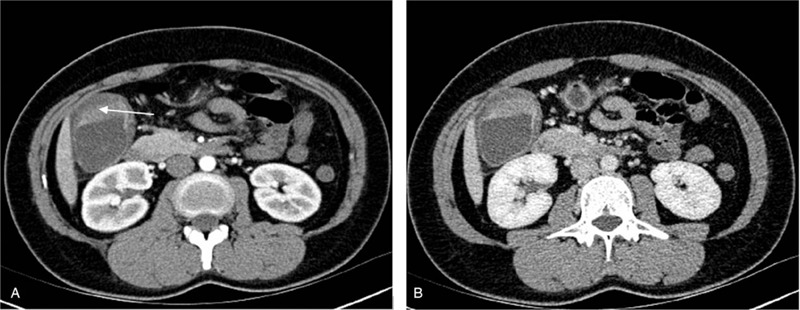
Typical contrast-enhanced CT images for GB-NEC. Continuous thin line-like contrast enhancement on the mucosa at (A) arterial and (B) venous phases. The arrow shows local wall thickening at the bottom of the gallbladder. CT = computed tomography, GB-NEC = gallbladder neuroendocrine carcinoma.

Six patients underwent contrast-enhanced CT examination, and 5 of them demonstrated the following imaging features: well-defined margins, and continuous thin line-like contrast enhancement on the mucosa (Fig. 1). Positron emission tomography-computer tomography (PET-CT) was performed for 2 patients: 1 patient showed lymph nodes, liver, gastric antrum, colon, and duodenum metastases, and the other showed lymph nodes, liver, and colon metastasis. Bone scan was performed for 1 patient and revealed bone metastasis. In addition, 5 patients underwent abdominal ultrasound, 2 patients underwent magnetic resonance cholangiopancreatography (MRCP), but no typical imaging features were revealed by these examinations.

All patients underwent liver function tests and infection tests. The positive rates were as followed: AST 14.3% (1/7), ALT 28.6% (2/7), ALP 57.1% (4/7), GGT 57.1% (4/7), albumin 57.1% (4/7), TBIL 0% (0/7), PT 14.3% (1/7), INR 42.9% (3/7), WBC 14.3% (1/7), and CRP 28.6% (2/7).

Serum tumor marker examination was preoperatively performed for all patients and the positive rate of CA-125, CA-199, CEA, AFP were 28.6% (2/7), 14.3% (1/7), 14.3% (1/7), 0% (0/7), respectively. Six patients underwent tumor marker examination during follow-up period and the positive rate were as followed: CA-125 83.3% (5/6), CA-199 16.7% (1/6), CEA 0.0% (0/6), AFP 0.0% (0/6). Another patient, who had not received surgical treatment, died half a month after diagnosis, and did not receive further examination and treatment.

### 3.3 Treatments

According to the preoperative examination results and intraoperative conditions, including local tumor infiltration, lymphatic metastasis, and distant metastasis, 6 patients underwent surgery. Two patients underwent laparoscopic radical cholecystectomy and postoperatively underwent chemotherapy with 2 and 7 cycles of cisplatin plus etoposide, respectively. Two patients underwent open radical cholecystectomy. One of them postoperatively underwent chemotherapy with 4 cycles of carboplatin plus etoposide, and the other patient refused further treatment. Due to the involvement of peripheral organs, 2 patients underwent open extended cholecystectomy. One patient just underwent tumor biopsy to confirm its pathological type, because bone metastasis was observed. None of these 3 patients underwent further chemotherapy. The specific treatment modalities were shown in Table 2.

**Table 2:**
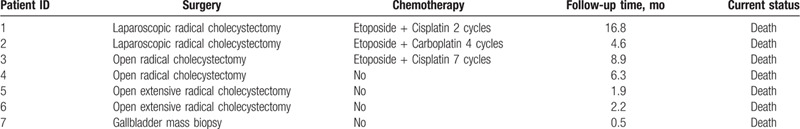
Treatment and follow-up of gallbladder neuroendocrine carcinoma.

### 3.4 Pathology and immunohistochemistry

We collected pathological specimens from all 7 patients, 6 were from surgical resection, and 1 was from tumor biopsy. All patients were diagnosed as poorly differentiated GB-NECs according to 2019 WHO classification. The positive rate of chromogranin A (CgA), synaptophysin (Syn), epithelial membrane (EMA), cytokine (CK) were 57.14% (4/7), 100% (7/7), 100% (3/3), 85.71% (6/7), respectively. Ki-67 index ranged from 2% to 80%, and mitotic count ranged from 25 to 124. According to postoperative pathological report, 1 patient was stage IIIA, and the other 6 patients were stage IVB. The specific date is listed in Table 3.

**Table 3:**
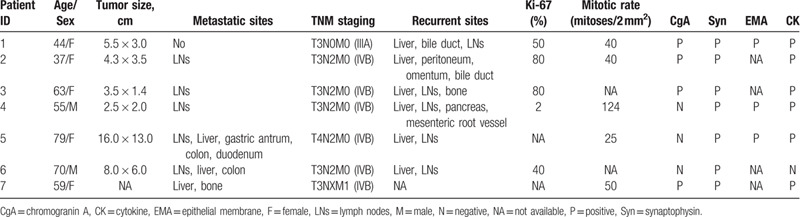
Pathological features of 7 gallbladder neuroendocrine carcinoma cases.

### 3.5 Postoperative outcomes

Two patients who underwent open extended cholecystectomy encountered bile leakage, and the drainage tubes removal days were 20 and 24 days, respectively. Both of them healed on their own by keeping drainage flowing. No patients encountered postoperative bleeding, abdominal cavity infection, gastrointestinal fistulas, or pulmonary complication. The average days of postoperative oral diet recovery, drainage tubes removal, and postoperative hospital stay were 6.3, 12.7, and 24.2 days, respectively. No patient underwent reoperation during postoperative hospitalization. The median overall survival of 7 patients was 4.6 months, (ranging from 0.5 to 16.8 months), and the 1-, 2-year survival rates were 14.29, 0% (Table 4).

**Table 4:**
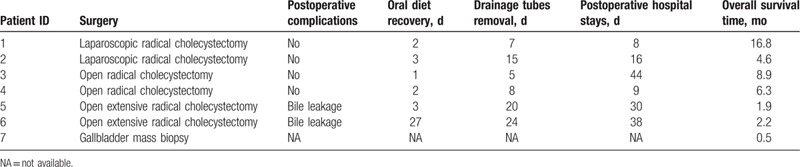
Surgery and postoperative outcomes of gallbladder neuroendocrine carcinoma.

### 3.6 Relationship between CA-125 and liver metastasis

Three patients presented with liver metastases before surgery, and 2 of them were CA-125 positive with the average of 51.5 U/mL. Among these 2 CA-125 positive patients, 1 patient underwent radical cholecystectomy, and the intrahepatic metastatic lesion was removed, and the CA-125 level returned to normal at 1 month after surgery. The other patient did not receive further examination and treatment, and died half a month after diagnosis. During the follow-up period, 5 patients presented with liver metastases, and the CA-125 level of all these patients elevated with the average of 68.3 U/mL (Table 5). None of these patients had ascites, ovarian carcinoma, peritoneal carcinoma, and endometrial carcinoma.

**Table 5:**
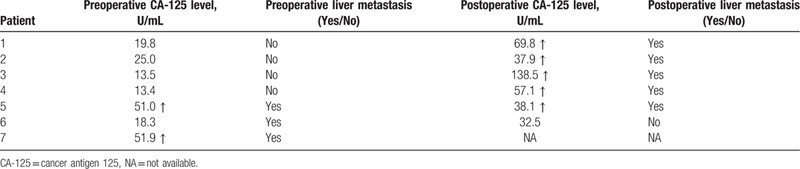
The level of CA-125 and liver metastasis of gallbladder neuroendocrine carcinoma.

## 4 Discussion

### 4.1 Epidemiology

Gallbladder carcinomas are the fifth most common gastrointestinal malignant tumors and adenocarcinomas are the most common histologic types.^[9]^ Other types include adenosquamous carcinomas, squamous carcinomas, and NECs. GB-NEC is very rare in clinical practice and there are few reports. According to SEER database, GB-NECs accounted for 2.1% of all gallbladder carcinomas.^[10]^ Kamboj et al^[11]^ reported the proportion of adenocarcinoma, NEC, squamous carcinoma, and adenosquamous carcinoma were 94.7%, 4.4%, 0.7%, and 0.2%, respectively. Most previous studies have found that GB-NEC is more common in middle-aged and elderly female patients. Chen et al^[12]^ showed the median age of 61 years with the male to female ratio of 1:4. In our study, the male to female ratio was 1:2.5 with a median age of 58 years (ranging from 37 to 79 years), which was consistent with most previous literatures.

### 4.2 Origin

There is no distribution of neuroendocrine cells in the gallbladder. We are aware that the pathogenesis of GB-NEC is controversial and still under investigation. Many researchers insist that GB-NEC originates from the intestinal or gastric metaplasia of gallbladder epithelium. Chronic inflammation is believed to promote metaplastic changes of gallbladder epithelial cells to neuroendocrine cells, since GB-NEC was mostly accompanied with cholelithiasis.^[4]^ In the study performed by Chen et al,^[12]^ 80% patients with GB-NEC had the history of cholecystitis. In our study, we observed that 3 of 7 patients had gallstones with cholecystitis. Some researchers propose that GB-NEC originates from the transformation of adenocarcinomas, since GB-NEC is often coexisted with adenocarcinomas. In addition, some researchers think that the neuroendocrine cells derive from local multipotent steam cells of gallbladder.^[13]^

### 4.3 Diagnosis

#### 4.3.1 Clinical manifestation

Functional GB-NEC is able to secret peptides such as serotonin and histamine which can cause specific symptoms, including diarrhea, flushing, edema, and wheezing. However, typical carcinoid syndrome is very rare (<1%).^[4]^ Jin et al^[14]^ reported a 65-year-old patient with GB-NEC presented with flushing and pathological specimens of the flushed skin showed that mucin was deposited between the collagen bundles in the dermis. In our study, no secretory symptoms were observed even in the patients with distant metastasis and upper abdominal pain was the most common symptom, consistent with most previous literatures.^[11,12,15]^

#### 4.3.2 Imaging and laboratory examination

Most studies indicated that GB-NEC lacks the typical imaging features that help distinguish it from other types of gallbladder carcinoma.^[12]^ However, in our study, 5 of 6 patients showed well-defined margins, and continuous thin line-like contrast enhancement were observed on the mucosa in contrast-enhanced CT examination. Kim et al^[16]^ retrospectively analyzed the contrast-enhanced CT differentiation of GB-NENs from adenocarcinomas and reported that well-defined margins were more often observed in GB-NENs than in GB-ADCs (94.7% vs 10.6%, *P* < .0001). Bae et al^[17]^ also reported that, compared with GB-ADCs, GB-NENs more frequently demonstrated well-defined margins, intact mucosa, and thick rim contrast enhancement and/or diffusion restriction in MRI examination. Researchers assume that microscopic location of NEN cells originate from the deep part of the lamina propria or submucosa, and thus the surface mucosal epithelium remained partially intact and was linearly enhanced. In addition, GB-NENs are more often manifested as gallbladder replacing type with larger hepatic and lymph node metastasis while adenocarcinomas as wall thickening type.^[16]^ It has been reported that PET-CT was effective for the detection of NENs and helpful for identification of distant metastasis.^[18,19]^ In our study, 2 patients underwent PET-CT examination which showed adjacent and/or distant metastasis, and the examination results are valuable for preoperatively determining the TNM staging and therapeutic strategy.

CA-125 is a tumor marker of ovarian carcinoma, peritoneal carcinoma, and endometrial carcinoma. However, CA-125 was routinely examined in our hospital, and we accidentally found that there may be a relationship between CA-125 and GB-NEC liver metastasis. In our study, among the patients with liver metastases before surgery, 66.7% of patients were CA-125 positive, and among the patients presented with liver metastases during follow-up, 100% of patients were CA-125 positive. In addition, in a patient with CA-125 positive before surgery, CA-125 level returned to normal after resection of intrahepatic metastases. However, during the follow-up period, this patient had liver metastasis again and CA-125 level also elevated. All patients with elevated CA-125 did not have ascites, ovarian carcinoma, peritoneal carcinoma, and endometrial carcinoma. Yun et al^[20]^ reported that serum CA-125 levels in the gallbladder carcinoma group were significantly higher when compared with benign gallbladder disease group and healthy control group. However, no literature had reported the relationship between Cancer antigen 125 (CA-125) elevation and liver metastasis of GB-NEC. We just conjecture that CA-125 elevation may indicate liver metastasis or tumor progression. More investigations are necessary to further confirm this relationship, which is valuable for preoperatively determining therapeutic strategy and evaluating prognosis.

### 4.4 Treatment

#### 4.4.1 Surgery

As a malignant tumor, surgical resection remains as the preferred and primary treatment for GB-NEC. The surgical procedures vary from simple cholecystectomy to extended cholecystectomy.^[21]^ It has been reported that simple cholecystectomy is enough for in situ GB-NENs and those of T1N0M0 stage.^[4]^ In the study performed by Liu et al,^[22]^ all 3 GB-NEC patients who were in T1bN0M0 stage underwent laparoscopic cholecystectomy with gallbladder bed cautery, and no recurrences were found without any chemotherapy or radiotherapy during the follow-up period of at least 26 months. For those who are diagnosed at more advanced stage without distant metastasis, radical cholecystectomy is the preferred treatment if it is available. Compared with other treatment modalities, radical resection could improve survival of patients with GB-NEC.^[23]^ In our study, the median survival time of 4 patients undergoing radical cholecystectomy and 3 patients undergoing other treatments were 7.6 and 1.9 months, respectively. With the improvement of surgical techniques, laparoscopic radical cholecystectomy is technically feasible and may be used for patients with advanced GB-NEC. Kim et al^[24]^ reported a patient with GB-NEC diagnosed at T3N1M0 stage underwent laparoscopic radical cholecystectomy and combined chemoradiation therapy, and no evidence of recurrence during the follow-up period of 14 months. In our study, 2 patients underwent laparoscopic radical cholecystectomy and combined chemotherapy. Both of them didn’t encounter bile leakage, pancreatic fistula, postoperative bleeding, abdominal cavity infection, gastrointestinal fistulas, or pulmonary complication, with the overall survival time of 4.6 and 16.8 months, respectively. For patients with distant metastasis, the value of surgery remains controversial. Palliative cholecystectomy may reduce tumor burden to facilitate the implementation of postoperative treatment and improve the quality of life.

#### 4.4.2 Chemotherapy

As most patients of GB-NECs diagnosed at advanced stage, many patients have lost the opportunity of radical surgery. Chemotherapy is critical for patients with unresectable GB-NECs, and can improve survival.^[12,15]^ In our study, compared with the patients who only received surgery, the patients who underwent postoperatively chemotherapy lived longer with a median survival time of 8.9 and 2.2 months, respectively. Cisplatin or carboplatin plus etoposide were generally recommended as the primary program for poorly differentiated GB-NECs and achieved satisfactory responses.^[25]^ In addition, Kanetkar et al^[26]^ reported that in carefully selected patients, new adjuvant chemotherapy can help to reduce tumor burden and facilitate radical resection with negative margins.

### 4.5 Prognosis

Due to the malignancy and delayed diagnosis of GB-NEC, the prognosis is poor. In our study, all patients were diagnosed at advanced stage, and the median overall survival was 4.6 months with 1-, 2-year survival rate of 14.29%, 0%. Kamboj et al^[11]^ showed a median survival time of 3 months (ranging from 1 to 9.5 months). According to SEER database,^[10]^ the median overall survival was 9.8 months among 278 cases of GB-NENs and 5-year survival rate in NET was 36.9%, while in NEC was 0%. Most previous studies reported that GB-NEC is associated with a worse prognosis than GB-ADC, which researchers believed that it was due to higher percentage patients with lymph node metastases and advanced stage. In the study performed by Duffy et al,^[9]^ the median survival time of 13 patients with GB-NEC was 9.8 months (95% CI 5.3–20.1 months), while of the entire gallbladder carcinomas was 10.3 months (95% CI 8.8–11.8 months). Chen et al^[12]^ reported a median survival time of 3 months with 1-, 2-, and 3-year survival rates of 20%, 10%, and 0%, while the median survival time of the 377 GB-ADC patients treated during the same period was 6 months with 1-, 2-, 3-, and 5-year survival rates of 38.0%, 31.0%, 30.1%, and 28.4%, respectively. However, Yun's study showed that the overall 5-year survival rate of GB-NEC was higher than GB-ADC.^[20]^ Suspecting that confounding factors such as age, sex, Nevin stage, and radical surgery between the 2 groups might affect the result, Yan et al^[15]^ used propensity score matching to reduce the influence of confounding factors between the two groups, and the results still showed that GB-NEC had a worse prognosis than GB-ADC.

Our study also has several limitations. Firstly, it was a retrospective study and the data collection was limited. Secondly, due to the rarity of GB-NEC, only 7 patients were included in the study, and the treatment of each patient was variable, which led to difficulty of statistical analysis. Therefore, prospective studies with larger samples are necessary to conduct to analyze clinical characteristics of GB-NEC and to find more appropriate diagnosis and treatments.

## 5 Conclusion

Surgical resection, including laparoscopic radical cholecystectomy, is feasible for the treatment of advanced GB-NEC in selected patients and has the advantages of prolonging survival in combination with chemotherapy. The elevation of CA-125 can be utilized as an important predictor of poor prognosis, while more investigations are necessary to confirm this relationship. It may be necessary to conduct a multicenter, well-designed study to assess the predictive effect of CA-125 levels on the oncological prognosis of GB-NEC.

## Author contributions

**Conceptualization:** Chengwu Zhang, Weiding Wu.

**Data curation:** Hongwu Chu, Chengwu Zhang.

**Formal analysis:** Hongwu Chu, Weiding Wu.

**Funding acquisition:** Jungang Zhang, Dongsheng Huang.

**Investigation:** Hongwu Chu.

**Methodology:** Hongwu Chu, Chengwu Zhang.

**Project administration:** Zhiming Hu, Jungang Zhang, Dongsheng Huang.

**Resources:** Jungang Zhang.

**Software:** Ying Shi, Weiding Wu.

**Validation:** Ying Shi, Zhiming Hu.

**Visualization:** Hongwu Chu, Ying Shi, Zhiming Hu.

**Writing – original draft:** Hongwu Chu.

**Writing – review & editing:** Jungang Zhang, Dongsheng Huang.
